# Supplementary material for: The DIAPH3 linker specifies a β-actin network that maintains RhoA and Myosin-II at the cytokinetic furrow
Source: Nat Commun. 2024 Jun 19;15:5250. doi: 10.1038/s41467-024-49427-2 (PMC11187180; doi:10.1038/s41467-024-49427-2)
Supplement: Supplementary file 1 — Supplementary Information [file 41467_2024_49427_MOESM1_ESM.pdf]

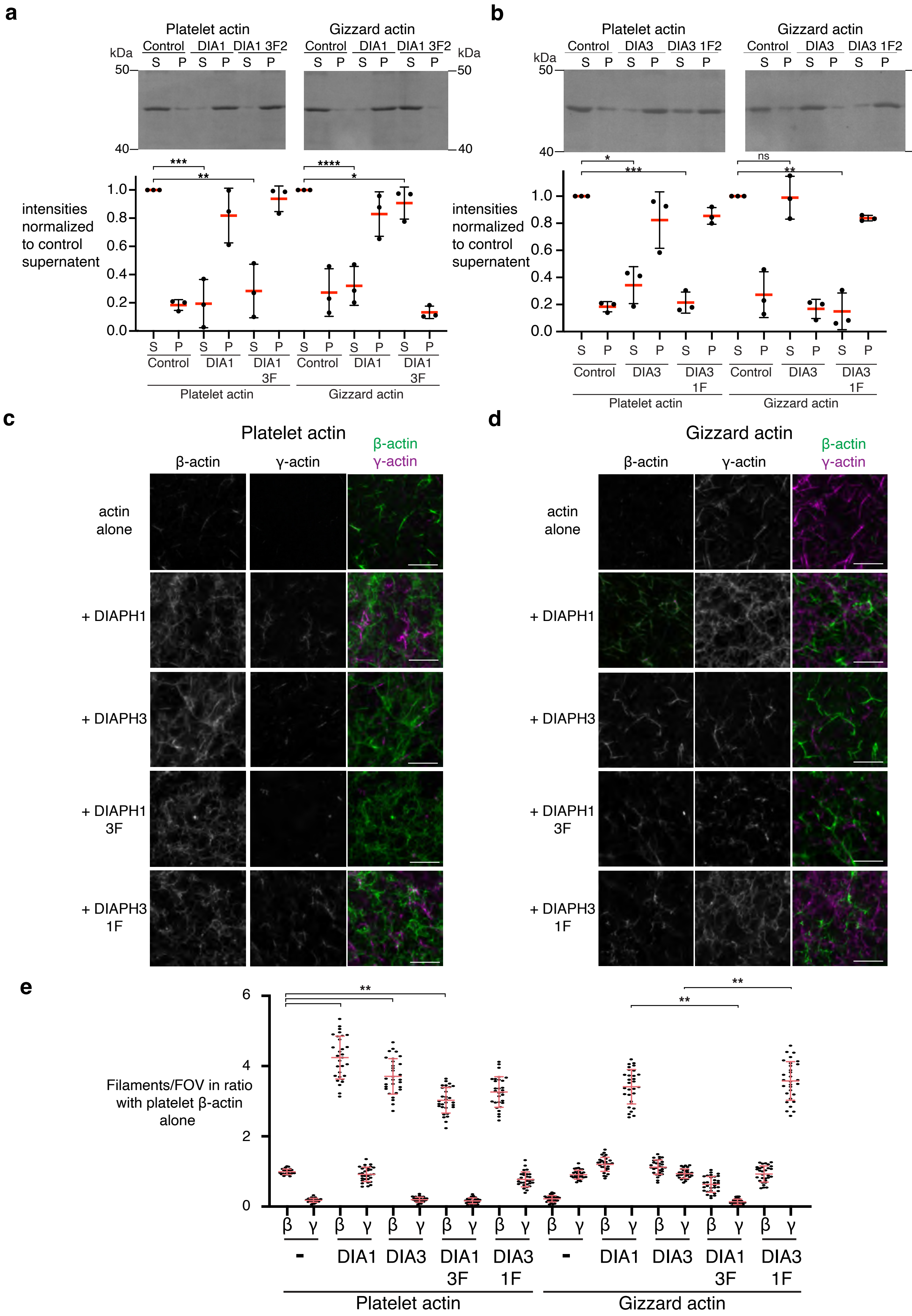

a
Platelet actin
Gizzard actin
Control
DIA1
DIA1 3F2
kDa
S P
50
40
intensities normalized to control supernatent
Platelet actin
Gizzard actin
b
Control
DIA3
DIA3 1F2
DIA3 1F
c
Platelet actin
β-actin
γ-actin
actin alone
+ DIAPH1
+ DIAPH3
+ DIAPH1 3F
+ DIAPH3 1F
d
Gizzard actin
e
Filaments/FOV in ratio with platelet β-actin alone
DIA1 3F

Supplementary Figure 1.

The FH2 domain of DIAPH3 specifically generates β-actin homopolymers. **a** Top: The CT of wildtype DIAPH1and DIAPH1 CT containing the DIAPH3 FH2 domain were fused to GST and incubated with actin purified from platelets (β-actin rich) or gizzard (γ-actin rich) and polymerized actin (P) separated from unpolymerized (S) by centrifugation. The fractions were then analyzed by SDS-PAGE and Coomassie blue staining. Gels representative of three independent experiments are presented. Bottom: Quantitation of band intensities for pelleting assay. N = 3; data are presented as mean (red bars) ± SD. *p = 0.029, **p = 0.027, ***p = 0.015, ****p = 0.014 as determined by two-sided Mann-Whitney non-parametric tests. **b** The CT of wildtype DIAPH3 and DIAPH3 containing the FH2 domain of DIAPH1 were fused to GST and incubated with actin purified from platelets (β-actin rich) or gizzard (γ-actin rich) and polymerized actin (P) separated from unpolymerized (S) by centrifugation. The fractions were then analyzed by SDS PAGE and Coomassie blue staining. Gels representative of three independent experiments are presented. Bottom: Quantitation of band intensities for pelleting assay. N = 3; data are presented as mean (red bars) ± SD. 'ns' = 0.91, *p = 0.014, **p = 8.35×10^{-3}, ***p =3.26×10^{-3} as determined by two-sided Mann-Whitney non-parametric tests. **c** and **d** Micrographs of the experiments outlined in A and B but analyzed by placing the reaction on glass coverslips then fixed and stained using antibodies that specifically recognize β- and γ-actin isoforms. **e** Quantitation of the number of filaments per field of view (FOV) from experiments outlined in panels C and D. n = 30 FOV analyzed per condition across three independent experiments, presented as mean (red bars) ± SD. *p = 1.00×10^{-15} as determined by two-sided Mann-Whitney non-parametric tests.

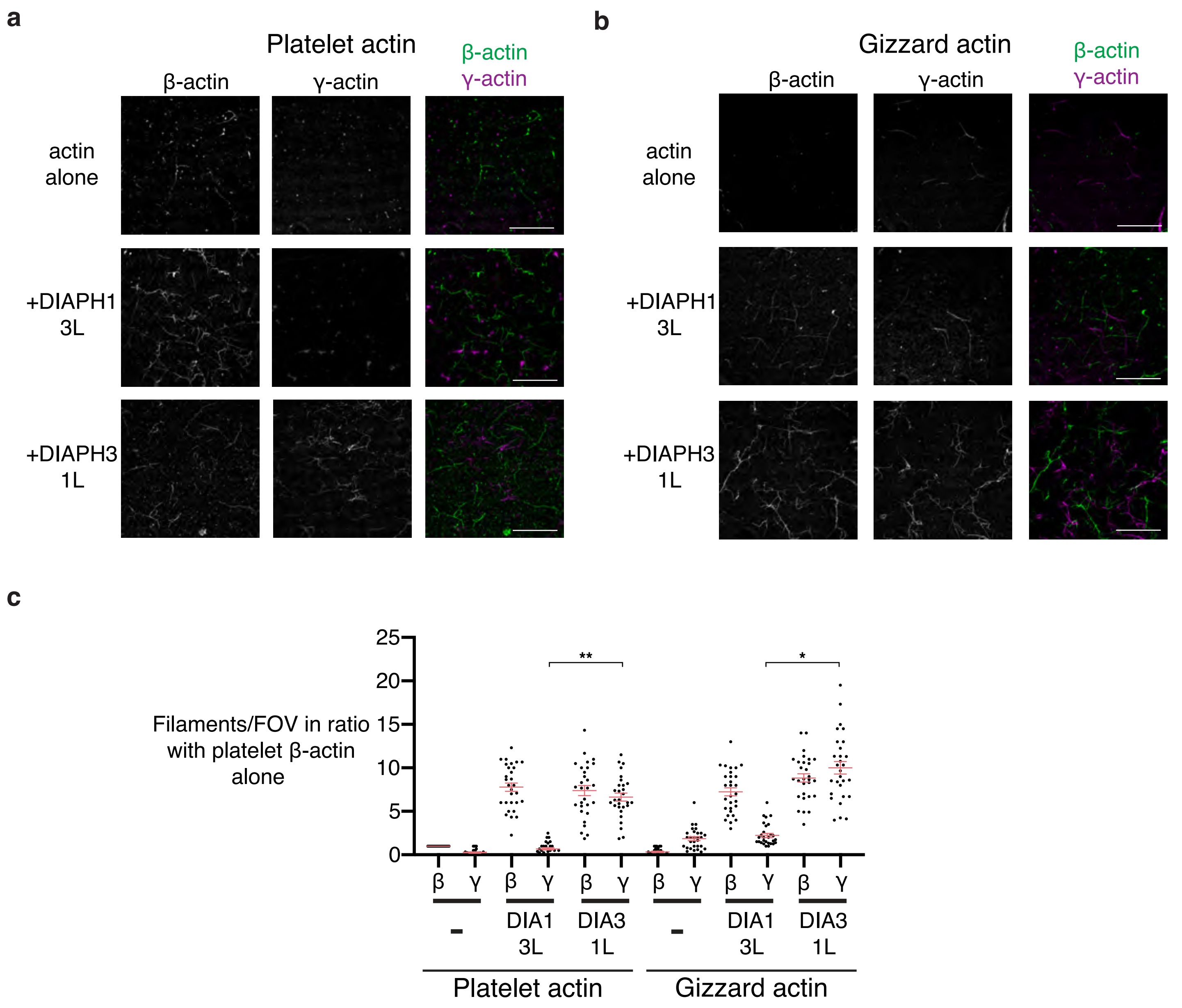

a
Platelet actin
β-actin
γ-actin
β-actin
γ-actin
actin alone
+DIAPH1 3L
+DIAPH3 1L
b
Gizzard actin
β-actin
γ-actin
β-actin
γ-actin
actin alone
+DIAPH1 3L
+DIAPH3 1L
c
Filaments/FOV in ratio with platelet β-actin alone
25
20
15
10
5
0
**
*
β
γ
-
DIA1 3L
DIA3 1L
Platelet actin
Gizzard actin

Supplementary Figure 2.

The linker region within the FH2 domain of DIAPH3 specifically generates β-actin homopolymers. The CT of wildtype DIAPH1, DIAPH1 containing the DIAPH3 linker region domain (DIAPH1-3L), wildtype DIAPH3 or DIAPH3 containing the linker region of DIAPH1 (DIAPH3-1L), were fused to GST and incubated with **a** actin purified from platelets (β-actin rich) and **b** actin purified from gizzard (γ-actin rich). The reactions were then spotted onto glass coverslips, fixed, and stained with actin isoform specific antibodies. **c** Quantification of the reactions in a and b. n = 30 FOV analyzed per condition across three independent experiments. Data are presented as mean ± SD. $^*p = 2.20 \times 10^{-14}$, $^{**}p = 1.00 \times 10^{-15}$ as determined by two-sided Mann-Whitney non-parametric tests.

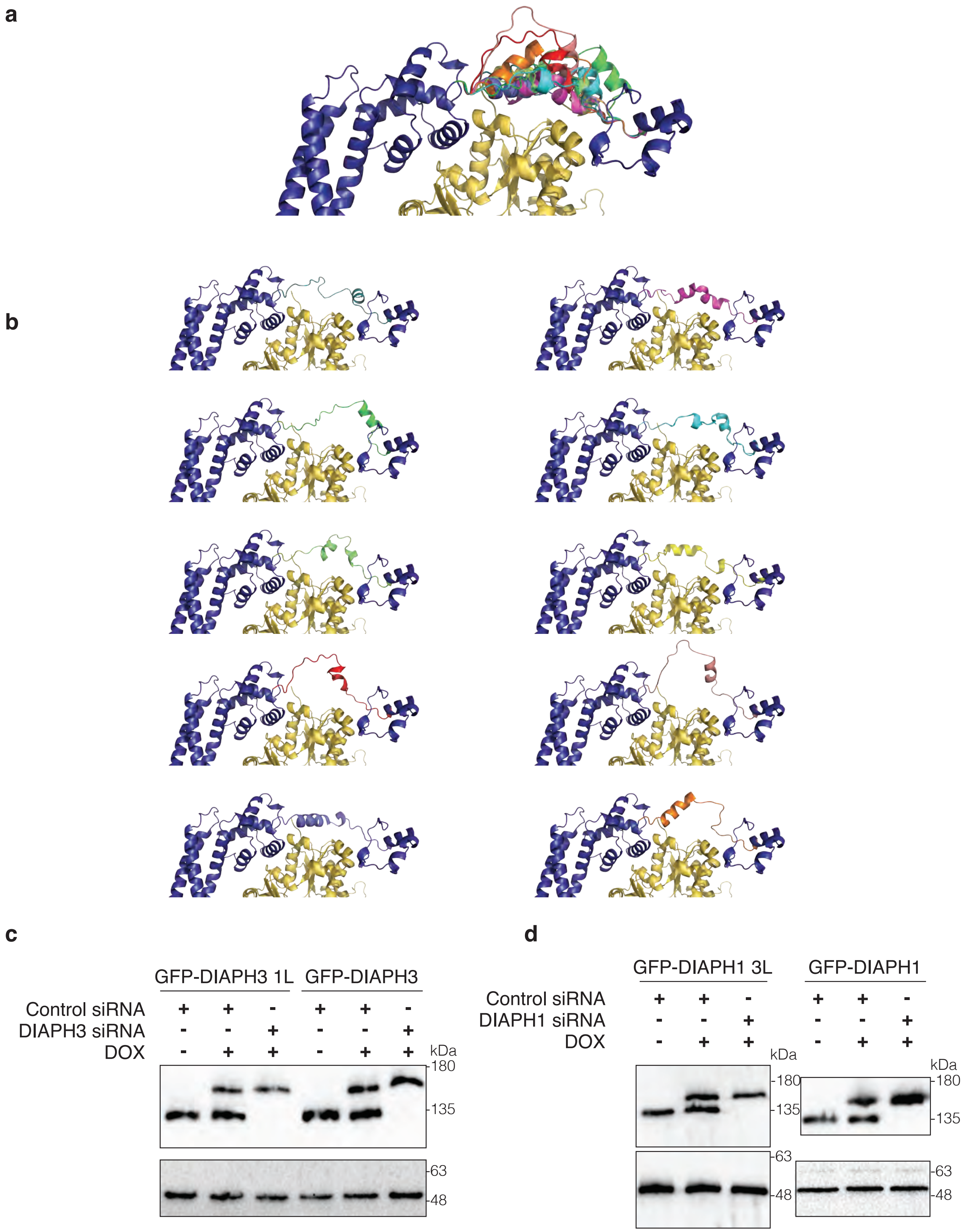
a
b
c
GFP-DIAPH3 1L
GFP-DIAPH3
Control siRNA + + - + + -
DIAPH3 siRNA - - + - - +
DOX - + + - + +
kDa
180
135
63
48
d
GFP-DIAPH1 3L
GFP-DIAPH1
Control siRNA + + - + + -
DIAPH1 siRNA - - + - - +
DOX - + + - + +
kDa
180
135
63
48

Supplementary Figure 3.

The DIAPH3 FH2 linker region is predicted to adopt multiple conformations. **a** The DIAPH3 FH2 domain (dark blue) modelled in complex with β-actin (yellow) with 10 LDRS generated DIAPH3 FH2 domain linker regions (color spectrum). **b** Linker region conformers from the ensemble in panel A presented individually in the same context. For both panels A and B, a single β-actin N-terminal conformer is shown for simplicity. **c** Lysates collected from stable HeLa cell lines inducibly-expressing GFP-DIAPH1-3L treated as indicated were subjected to SDS-PAGE and immunoblotting with anti-DIAPH1 and anti-α-tubulin antibodies. The western blot presented is representative of two independent experiments. **d** Lysates collected from stable HeLa cell lines inducibly-expressing GFP-DIAPH3-1L treated as indicated were subjected to SDS-PAGE and immunoblotting with anti-DIAPH3 and anti-α-tubulin antibodies. The western blot presented is representative of two independent experiments.

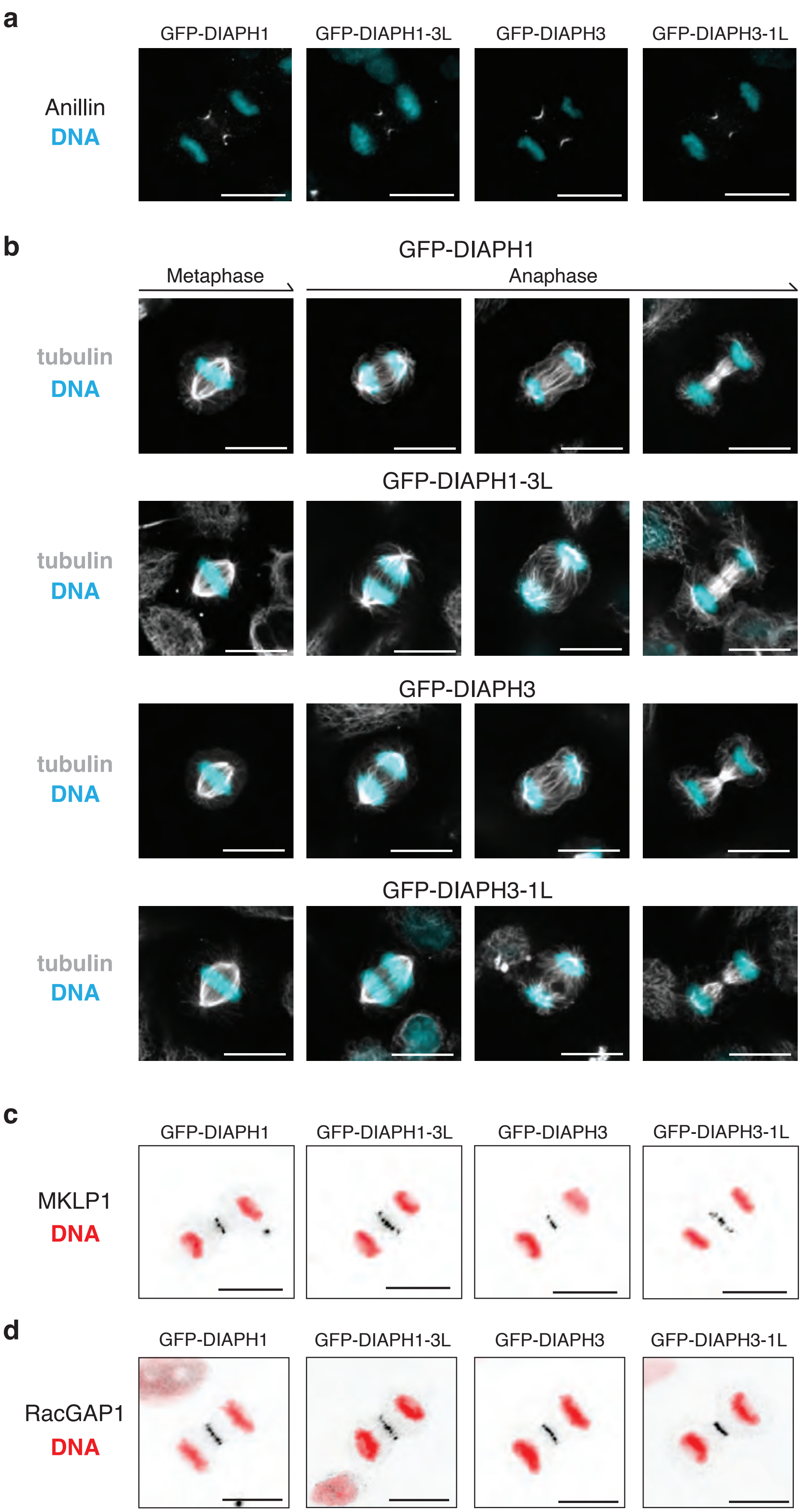
a
GFP-DIAPH1
GFP-DIAPH1-3L
GFP-DIAPH3
GFP-DIAPH3-1L
Anillin
DNA
b
GFP-DIAPH1
Metaphase
Anaphase
tubulin
DNA
GFP-DIAPH1-3L
tubulin
DNA
GFP-DIAPH3
tubulin
DNA
GFP-DIAPH3-1L
tubulin
DNA
c
GFP-DIAPH1
GFP-DIAPH1-3L
GFP-DIAPH3
GFP-DIAPH3-1L
MKLP1
DNA
d
GFP-DIAPH1
GFP-DIAPH1-3L
GFP-DIAPH3
GFP-DIAPH3-1L
RacGAP1
DNA

Supplementary Figure 4.

Anillin, microtubule, MKLP1, and RacGAP1 localization are unaffected by changes in actin isoform network redistribution. HeLa cells expressing different DIAPH1 and DIAPH3 variants with altered actin isoform specificity were fixed and stained with **a** an anillin antibody (DNA in blue), **b** an α-tubulin antibody (DNA in blue), **c** a MKLP1 antibody (antibody staining in black, DNA in red) and **d** a RacGAP1 antibody (antibody staining in black, DNA in red). Scale bars in all panels represent 10μm. All micrographs displayed are representative of three independent experiments.

a

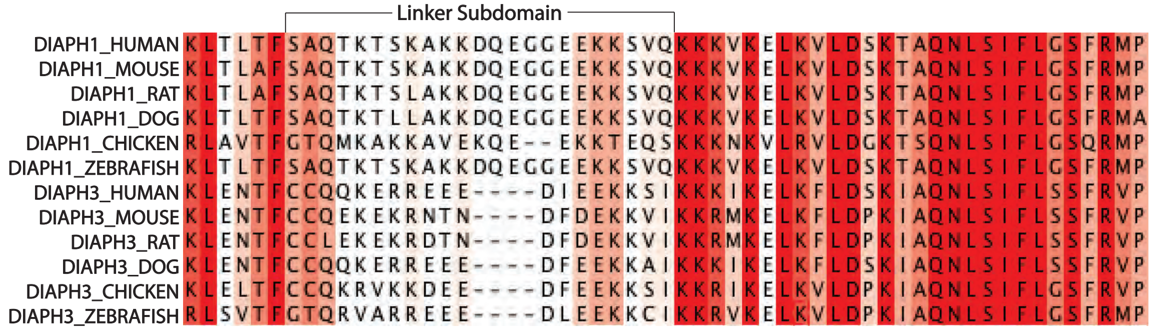

b

Formin	Length	Net Charge
DIAPH1_HUMAN	**23**	**+1**
DIAPH1_MOUSE	**23**	**+1**
DIAPH1_RAT	**23**	**+0.5**
DIAPH1_DOG	**23**	**+0.5**
DIAPH1_CHICKEN	**21**	**+1.5**
DIAPH1_ZEBRAFISH	**23**	**+1**
DIAPH3_HUMAN	**19**	**-2**
DIAPH3_MOUSE	**19**	**-0.5**
DIAPH3_RAT	**19**	**-1.5**
DIAPH3_DOG	**19**	**-2.5**
DIAPH3_CHICKEN	**19**	**-0.5**
DIAPH3_ZEBRAFISH	**19**	**-1.5**

Supplementary Figure 5.

Comparison of different DIAPH1 and DIAPH3 linker regions. **a** Cross-species sequence alignment of different vertebrate DIAPH1 and 3 linker sequences. Darker shades of red indicate a greater degree of conservation. **b** Comparison of the linker region length and charge of DIAPH1 and 3 across different vertebrate sequences.

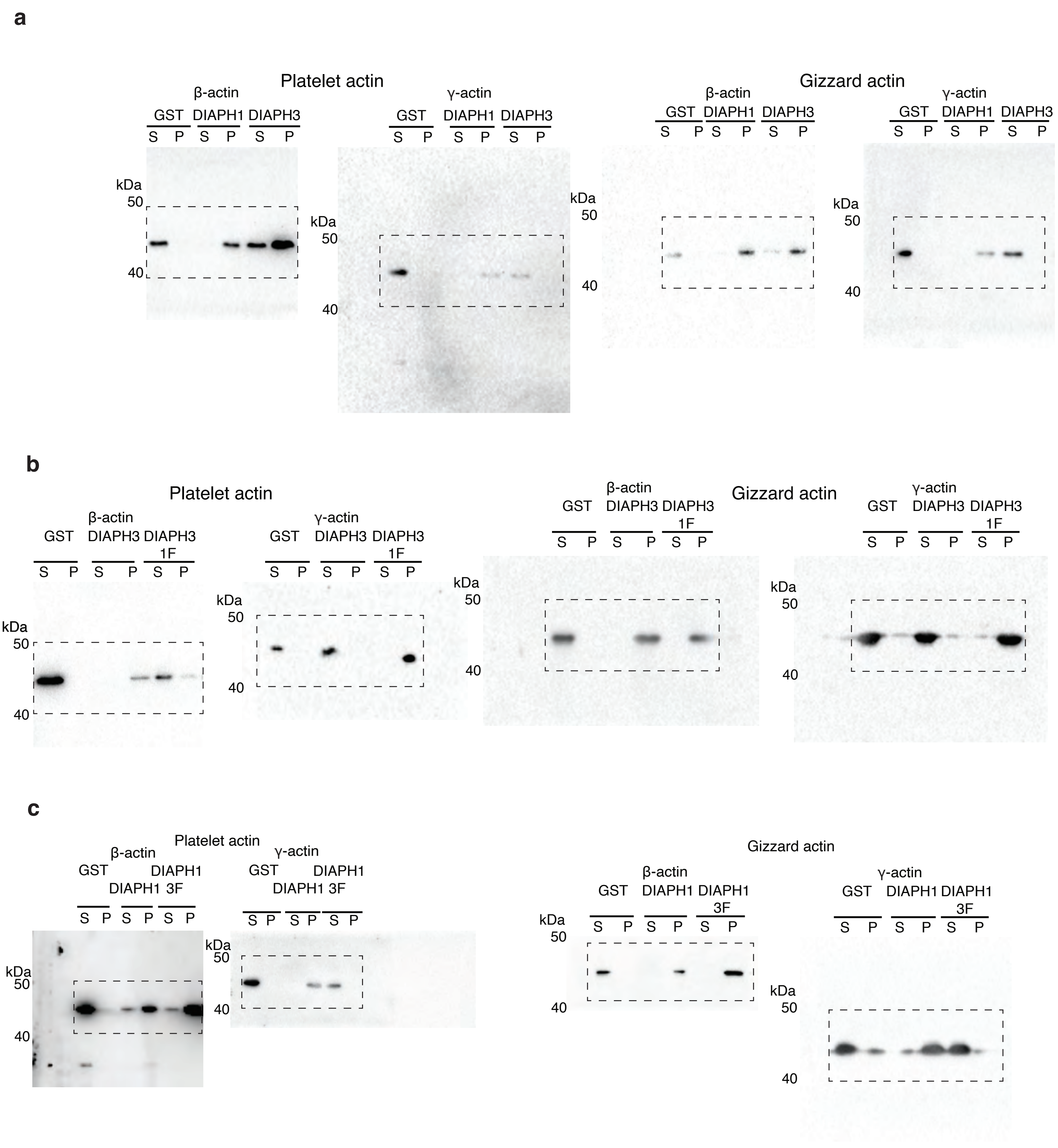
a
Platelet actin
β-actin
GST DIAPH1 DIAPH3
S P S P S P
kDa
50
40
γ-actin
GST DIAPH1 DIAPH3
S P S P S P
kDa
50
40
Gizzard actin
β-actin
GST DIAPH1 DIAPH3
S P S P S P
kDa
50
40
γ-actin
GST DIAPH1 DIAPH3
S P S P S P
kDa
50
40
b
Platelet actin
β-actin
GST DIAPH3 DIAPH3 1F
S P S P S P
kDa
50
40
γ-actin
GST DIAPH3 DIAPH3 1F
S P S P S P
kDa
50
40
Gizzard actin
β-actin
GST DIAPH3 DIAPH3 1F
S P S P S P
kDa
50
40
γ-actin
GST DIAPH3 DIAPH3 1F
S P S P S P
kDa
50
40
c
Platelet actin
β-actin
GST DIAPH1 DIAPH1 3F
S P S P S P
kDa
50
40
γ-actin
GST DIAPH1 DIAPH1 3F
S P S P S P
kDa
50
40
Gizzard actin
β-actin
GST DIAPH1 DIAPH1 3F
S P S P S P
kDa
50
40
γ-actin
GST DIAPH1 DIAPH1 3F
S P S P S P
kDa
50
40

Supplementary Figure 6

Uncropped blots associated with Figure 2. **a** Uncropped blots associated with Figure 2a. **b** Uncropped blots associated with Figure 2b. **c** Uncropped blots associated with Figure 2c.

a

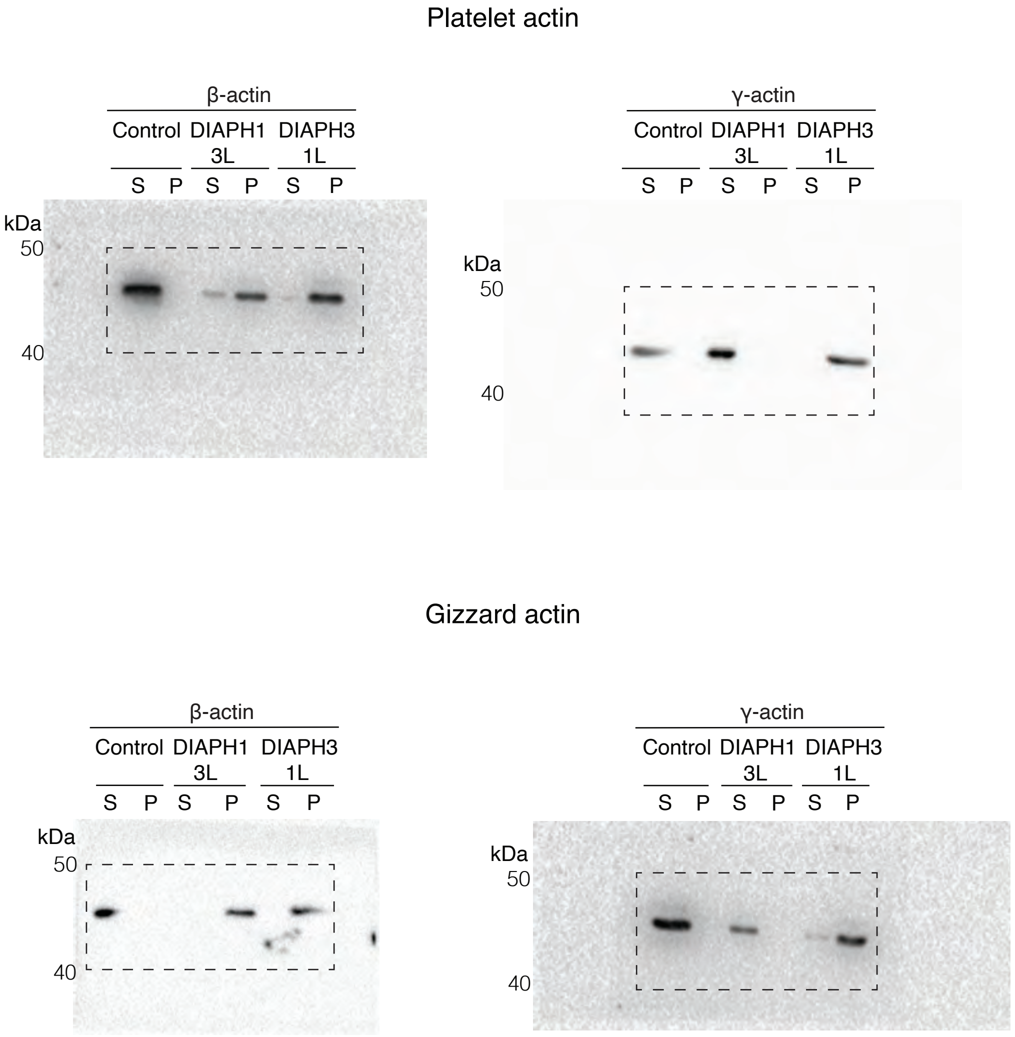
Platelet actin
β-actin
Control
DIAPH1
3L
DIAPH3
1L
S P S P S P
kDa
50
40
γ-actin
Control
DIAPH1
3L
DIAPH3
1L
S P S P S P
kDa
50
40
Gizzard actin
β-actin
Control
DIAPH1
3L
DIAPH3
1L
S P S P S P
kDa
50
40
γ-actin
Control
DIAPH1
3L
DIAPH3
1L
S P S P S P
kDa
50
40

Supplementary Figure 7

Uncropped blots associated with Figure 3. **a** Uncropped blots associated with Figure 3.

a

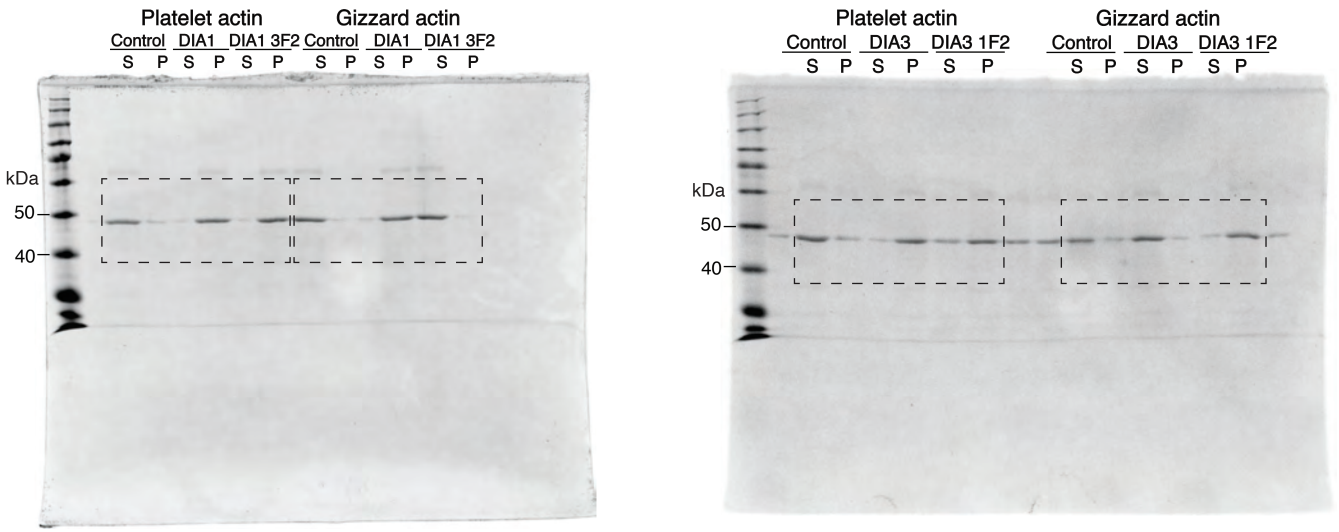
Platelet actin
Gizzard actin
Control
DIA1
DIA1 3F2
Control
DIA1
DIA1 3F2
S P S P S P S P S P S P
kDa
50
40
Platelet actin
Gizzard actin
Control
DIA3
DIA3 1F2
Control
DIA3
DIA3 1F2
S P S P S P S P S P S P
kDa
50
40

Supplementary Figure 8

Uncropped SDS-PAGE gels associated with Supplementary Figure 1. **a** Uncropped SDS-PAGE gels associated with Supplementary Figure 1.

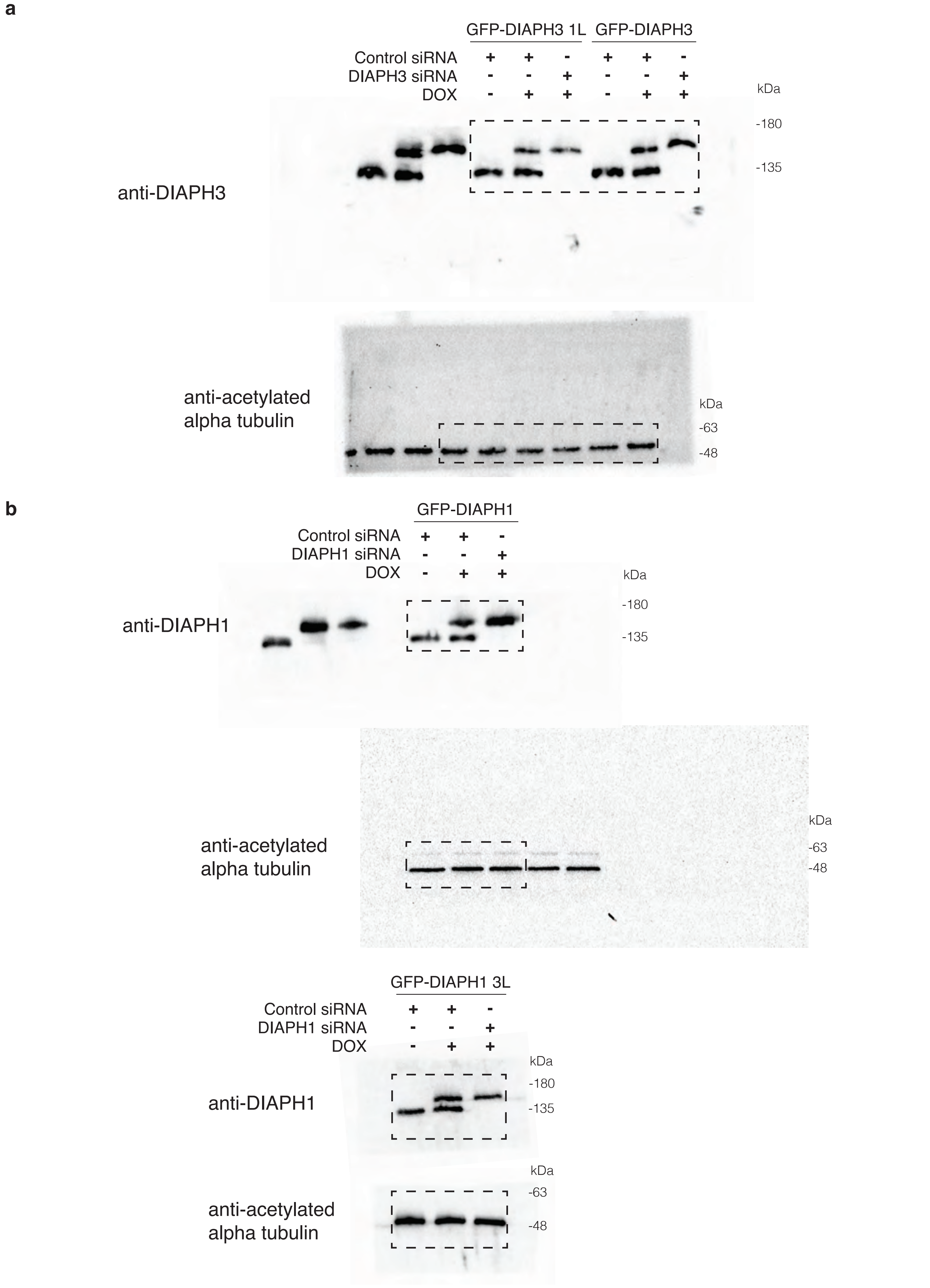
a
GFP-DIAPH3 1L
GFP-DIAPH3
Control siRNA + + - + + -
DIAPH3 siRNA - - + - - +
DOX - + + - + +
kDa
-180
-135
anti-DIAPH3
anti-acetylated alpha tubulin
kDa
-63
-48
b
GFP-DIAPH1
Control siRNA + + -
DIAPH1 siRNA - - +
DOX - + +
kDa
-180
-135
anti-DIAPH1
anti-acetylated alpha tubulin
kDa
-63
-48
GFP-DIAPH1 3L
Control siRNA + + -
DIAPH1 siRNA - - +
DOX - + +
kDa
-180
-135
anti-DIAPH1
kDa
-63
-48
anti-acetylated alpha tubulin

Supplementary Figure 9

Uncropped blots associated with Supplementary Figure 3. **a** Uncropped blots associated with Supplementary Figure 3a. **b** Uncropped blots associated with Supplementary Figure 3b.

**Primers to make**	**Primer Sequences (5' --> 3') [fwd top; rev bottom]**
Tom20-GFP DIAPH1 CT	ATGGACGAGCTGTACAAGAGTGCTGCTGTTCCCC
	TCTAGAGTCGCGGCCGCTTTAGCTTGCACGGCCAACC
Tom20-GFP DIAPH3 CT	ATGGACGAGCTGTACAAGAAACTAGAAGAGTTTGAAGAGAAAGCA
	TCTAGAGTCGCGGCCGCTTTATAAAGCTCGTAATCTTGCCAGCAG
pGEX-6P-2 DIAPH1 CT	TTCCAGGGGCCCCTGGGATCCAGTGCTGCTGTTCCCCCT
	TCAGTCACGATGCGGCCGCTTAGCTTGCACGGCCAACC
pGEX-6P-2 DIAPH3 CT	TTCCAGGGGCCCCTGGGATCCAAACTAGATGAGTGGGAAGAG
	TCAGTCACGATGCGGCCGCTTATAAAGCTCGTAATCTTGC
pcDNA5/TO DIAPH1 FL	CCATTAATTAAGGATCCAATGGAGCCGTCCGGCGGG
	GCATATCTGCAGAATTCCTTAGCTTGCACGGCCAACCAGC
pcDNA5/TO DIAPH3 FL	CCATTAATTAAGGATCCAATGGAGAGGCACCGGGCG
	GCATATCTGCAGAATTCTTATAAAGCTCGTAATCTTGCCAG
DIAPH1 3L	CAAAGTCATTTGTATTCCTTTTCTCTTTTTCTTGGCAACAGAAGGCAAGGGTAAGTTTGG
	AAAAGGAATACAAATGACTTTGATGAGAAGAAAGTTATTAAGAAGAAAGTAAAAGAGCTG
DIAPH3 1L	CTTGATCCTTCTTGGCTTTAGAAGTCTTGGTCTGGGCGGAAAAAGTGTTCTCAAGTTTAC
	AGAAGGATCAAGAAGGTGGAGAAGAAAAGAAATCTGTTCAAAAGAAGAGAATGAAGGAAC
DIAPH1 3F2	GCGGCCCCAGTTCTGCCATTTGGTTTGAAACCAAAGAAAGAA
	CTCCAGTCGCTCTTTTTCTGCTCGCTCTTTCGCTATCCTGGC
DIAPH3 1F2	TCTATTCCATTAAACCTGCCATTTGGATTAACCCCCAAAAAA
	TTCAAGTCGCTCTTTCTCTGCCTTCTCCTTGGCTAATTTTGC

Supplementary Table 1. List of oligonucleotides used in this study

Target	siRNA Duplex Sequences
3’UTR DIAPH1	5’-AAUAGCUGGGGCUUGUAAACCUTT-3’
	5’-GUUUCAAUGCUUUAUUAACAGUUGGAA-3’
3’UTR DIAPH3	5’-AAUAGCUGGGGCUUGUAAACCUTT-3’
	5’-GUUUCAAUGCUUUAUUAACAGUUGGAA-3’

Supplementary Table 2. List of siRNAs used in the study.

Target	Manufacturer	Catalog #	Species/ Isotype	Stock Concentration	Fixation	IF-Dilution Factor	WB - Dilution Factor
β-actin	BioRad	MCA5775GA	Mouse/ IgG1	1 mg/mL	PFA/ MeOH	500	600
γ-actin	BioRad	MCA5776GA	Mouse/ IgG2b	1 mg/mL	PFA/ MeOH	500	600
DIAPH3	Abcam	ab245660	Rabbit/ polyclonal	1 mg/mL	PFA	100	1000
DIAPH1	BD Biosciences	610848	Mouse/ IgG1	0.25 mg/mL	PFA	100	1000
Phospho-Myosin Light Chain 2 (Ser19)	Cell Signaling	3671	Rabbit/ polyclonal	0.1 mg/mL	PFA	50	N/A
RhoA	Santa Cruz	sc-179	Rabbit/ polyclonal	0.1 mg/mL	TCA	500	N/A
Ect2	Invitrogen	PA5-111074	Rabbit/ polyclonal	3.76 mg/mL	MeOH	100	N/A
MKLP1	Santa Cruz	sc-390113	Mouse/ IgG1	0.2 mg/mL	MeOH	500	N/A
Anillin	Santa Cruz	sc-271814	Mouse/ IgG2b	0.2 mg/mL	TCA	200	N/A
α-tubulin	Sigma	T6199	Mouse/ IgG1	1 mg/mL	MeOH	600	1000
RacGAP1	Novus Biologicals	NB100-884	Goat/ polyclonal	0.5 mg/mL	PFA	200	N/A
Anti-mouse IgG1 Alexa Fluor 488	Thermo Fisher	A28175	Goat	1 mg/mL	All	600	N/A
Anti-mouse IgG2b Alexa Fluor 488	Thermo Fisher	A21145	Goat	1 mg/mL	All	600	N/A
Anti-rabbit IgG Alexa Fluor 594	Thermo Fisher	A11012	Goat	1 mg/mL	All	400	N/A

Supplementary Table 3. The working concentrations and sources of antibodies used in this study.
