# Supplementary material for: The DIAPH3 linker specifies a β-actin network that maintains RhoA and Myosin-II at the cytokinetic furrow
Source: Nat Commun. 2024 Jun 19;15:5250. doi: 10.1038/s41467-024-49427-2 (PMC11187180; doi:10.1038/s41467-024-49427-2)
Supplement: Supplementary file 3 — Reporting Summary [file 41467_2024_49427_MOESM3_ESM.pdf]

nature portfolio

Corresponding author(s): Andrew Wilde

Last updated by author(s): May 20, 2024

Reporting Summary

Nature Portfolio wishes to improve the reproducibility of the work that we publish. This form provides structure for consistency and transparency in reporting. For further information on Nature Portfolio policies, see our Editorial Policies and the Editorial Policy Checklist.

Statistics

For all statistical analyses, confirm that the following items are present in the figure legend, table legend, main text, or Methods section.

n/a	Confirmed	
☐	☒	The exact sample size (*n*) for each experimental group/condition, given as a discrete number and unit of measurement
☐	☒	A statement on whether measurements were taken from distinct samples or whether the same sample was measured repeatedly
☐	☒	The statistical test(s) used AND whether they are one- or two-sided<br>*Only common tests should be described solely by name; describe more complex techniques in the Methods section.*
☒	☐	A description of all covariates tested
☒	☐	A description of any assumptions or corrections, such as tests of normality and adjustment for multiple comparisons
☐	☒	A full description of the statistical parameters including central tendency (e.g. means) or other basic estimates (e.g. regression coefficient) AND variation (e.g. standard deviation) or associated estimates of uncertainty (e.g. confidence intervals)
☐	☒	For null hypothesis testing, the test statistic (e.g. *F*, *t*, *r*) with confidence intervals, effect sizes, degrees of freedom and *P* value noted<br>*Give P values as exact values whenever suitable.*
☒	☐	For Bayesian analysis, information on the choice of priors and Markov chain Monte Carlo settings
☒	☐	For hierarchical and complex designs, identification of the appropriate level for tests and full reporting of outcomes
☒	☐	Estimates of effect sizes (e.g. Cohen's *d*, Pearson's *r*), indicating how they were calculated

Our web collection on statistics for biologists contains articles on many of the points above.

Software and code

Policy information about availability of computer code

Data collection	N/A.
Data analysis	Metamorph v.7.6.5.0 (Molecular Devices), Autoquant X3.1.1. (Media Cybernetics), Adobe Photoshop v.23.5.5 (Adobe Inc), Adobe Illustrator v.27.9.1 (Adobe Inc), Image Lab v.6.1.0 (Bio-Rad), Microsoft Excel v16.66.1 (Microsoft), Microsoft Word v16.66.1 (Microsoft), Adobe Acrobat Pro 2023 (Adobe Inc), GraphPad Prism v10.0.2 (GraphPad Inc), Fiji v2.9.0, AlphaFold2 (Deepmind), IDPConformerGenerator v0.7.18, Google Colab (Google), PyMOL Molecular Graphics System v2.4.1 (Schrödinger, LLC), CollabFold v1.5.5 (Alphafold2)

For manuscripts utilizing custom algorithms or software that are central to the research but not yet described in published literature, software must be made available to editors and reviewers. We strongly encourage code deposition in a community repository (e.g. GitHub). See the Nature Portfolio guidelines for submitting code & software for further information.

Data

Policy information about availability of data

All manuscripts must include a data availability statement. This statement should provide the following information, where applicable:

- Accession codes, unique identifiers, or web links for publicly available datasets
- A description of any restrictions on data availability
- For clinical datasets or third party data, please ensure that the statement adheres to our policy

All data is available in the main text or the supplementary materials. Any plasmids and cell lines generated during and/or analysed during the current study are available from the corresponding author on reasonable request.

Research involving human participants, their data, or biological material

Policy information about studies with human participants or human data. See also policy information about sex, gender (identity/presentation), and sexual orientation and race, ethnicity and racism.

Reporting on sex and gender	N/A
Reporting on race, ethnicity, or other socially relevant groupings	N/A
Population characteristics	N/A
Recruitment	N/A
Ethics oversight	N/A

Note that full information on the approval of the study protocol must also be provided in the manuscript.

Field-specific reporting

Please select the one below that is the best fit for your research. If you are not sure, read the appropriate sections before making your selection.

[x] Life sciences [ ] Behavioural & social sciences [ ] Ecological, evolutionary & environmental sciences

For a reference copy of the document with all sections, see nature.com/documents/nr-reporting-summary-flat.pdf

Life sciences study design

All studies must disclose on these points even when the disclosure is negative.

Sample size	No statistical tests were performed to predetermine samples sizes. For all experiments, data were collected across at least three independent experiments (the well-established field standard) and assessed for statistically significant differences. Given the (i) consistent close agreement of measured values for treatment conditions across biological replicates; and (ii) the fact that (i) obtained for all treatment conditions across all experiments conducted in the present study; and (iii) the high degree of statistical significance observed between treatment conditions expected to differ; the sample sizes are reasoned to be sufficient to derive biologically-meaningful conclusions.
Data exclusions	In all in cellula fixed cell analyses, no data were excluded. In live-cell imaging analysis monitoring furrow ingression, cell elongation, and blebbing, cells that underwent apoptosis during anaphase induced by photo-toxicity were excluded from subsequent analyses.
Replication	For all analyses, in cells both fixed and live, as well as in vitro, at least three independent experiments were performed for each measured condition. All attempts at replication were successful, save for some instances of photo-toxicity induced cell death in live imaging experiments (these cells were omitted from analysis). For live-imaging experiments, at least 5 cells over at least three independent experiments were analyzed.
Randomization	To compare the effects of manipulations on stable cell lines, all differentially treated cells were split from the same parental batch at the same time. Efforts were taken by analyzers of fixed samples to randomly select one slide among many (corresponding to all experimental conditions of the given experiment) to count/score/measure without prior knowledge of its treatment condition identity. Visualized regions of coverslips were chosen at random, but guarded against overlapping by altering the stage position with a choreographed pattern. For live-cell imaging analyses, the number of cells observed/analyzed were not predetermined. Cells were selected on the basis of their exhibiting the proper cell cycle stage (rounded mitotic cell) as found by randomly sampling FOVs.
Blinding	All experiments performed included control groups to examine the effects of a variable across different treatment conditions, thus blinding is not applicable to this study.

Reporting for specific materials, systems and methods

We require information from authors about some types of materials, experimental systems and methods used in many studies. Here, indicate whether each material, system or method listed is relevant to your study. If you are not sure if a list item applies to your research, read the appropriate section before selecting a response.

Materials & experimental systems

n/a	Involved in the study
☐	☒ Antibodies
☐	☒ Eukaryotic cell lines
☒	☐ Palaeontology and archaeology
☒	☐ Animals and other organisms
☒	☐ Clinical data
☒	☐ Dual use research of concern
☒	☐ Plants

Methods

n/a	Involved in the study
☒	☐ ChIP-seq
☒	☐ Flow cytometry
☒	☐ MRI-based neuroimaging

Antibodies

Antibodies used	Antibodies used in the study are presented below, in the format of "target (manufacturer, catalogue number, species/isotype)". Further information can be found in Supp Table 3 and the websites of each antibody's manufacturer.<br>β-actin (BioRad, MCA5775GA, Mouse/IgG1), γ-actin (BioRad,MCA5776GA, Mouse/IgG2b)<br>DIAPH3 (Abcam, ab245660, Rabbit/polyclonal), DIAPH1 (BD Biosciences, 610848, Mouse/IgG1),<br>Phospho-Myosin Light Chain 2 (Ser19) (Cell Signaling, 3671, Rabbit/polyclonal) RhoA (Santa Cruz, sc-179, Rabbit/polyclonal), Ect2 (Invitrogen, PA5-111074, Rabbit/polyclonal), MKLP1 (Santa Cruz, sc-390113, Mouse/IgG1), Anillin (Santa Cruz, sc-271814, Mouse/IgG2b), α-tubulin(Sigma, T6199, Mouse/IgG1), RacGAP1 (Novus Biologicals, NB100-884, Goat/polyclonal), Anti-mouse IgG1 Alexa Fluor 488 (ThermoFisher, A28175, Goat) Anti-mouse IgG2b Alexa Fluor 488 (ThermoFisher, A21145, Goat), Anti-rabbit IgG Alexa Fluor 594 (ThermoFisher, A11012, Goat)
Validation	All antibodies are commercially available and validated by the manufacturer as described on their respective websites.

Eukaryotic cell lines

Policy information about cell lines and Sex and Gender in Research

Cell line source(s)	HeLA FRT/TO (female) obtained from Dr. Laurence Pelletier, University of Toronto
Authentication	Cells were authenticated on the basis of exhibiting zeocin and blasticidin resistance as expected of HeLA FRT/TO cells.
Mycoplasma contamination	Cells were regularly tested and found negative for mycoplasma.
Commonly misidentified lines (See ICLAC register)	No such cells were used in this study.

Plants

Seed stocks	N/A
Novel plant genotypes	N/A
Authentication	N/A
